# A new species of *Thrombasia* J.L. Barnard, 1966 (Crustacea, Amphipoda, Tryphosidae) from the Clarion-Clipperton Zone, Central Pacific Ocean

**DOI:** 10.3897/zookeys.1274.140063

**Published:** 2026-03-24

**Authors:** Tammy Horton, Georgina Valls Domedel, Ed A. Hendrycks

**Affiliations:** 1 National Oceanography Centre, Southampton, SO14 3ZH, UK National Oceanography Centre Southampton United Kingdom; 2 Canadian Museum of Nature, Research Associate, Research and Collections, P.O. Box 3443, Station D, Ottawa, K1P 6P4, Canada Canadian Museum of Nature, Research Associate, Research and Collections Ottawa Canada

**Keywords:** Abyss, amphipods, Clarion-Clipperton Zone, deep sea, identification key, Lysianassoidea, Pacific Ocean, systematics

## Abstract

A new species of the genus *Thrombasia* within the family Tryphosidae is described from the Clarion-Clipperton Zone in the Pacific Ocean. A key to distinguish the new species from the six known species is provided, as well as the first molecular barcodes for the genus.

## Introduction

The amphipod genus *Thrombasia* J.L. Barnard, 1966 is included within the subfamily Tryphosinae and belongs to a subset of taxa known as the ‘Tryphosa group’ as defined by Kilgallen and Lowry (2014), consisting of eight genera: (*Bruunosa* Barnard & Karaman, 1987; *Glorieusella* Kilgallen & Lowry, 2014, *Gronella* Barnard & Karaman, 1991, *Metambasia* Stephensen, 1923, *Pseudonesimus* Chevreux, 1926, *Schisturella* Norman, 1900, *Thrombasia* J.L. Barnard, 1966 and *Tryphosa* Boeck, 1871) which are defined by the possession of a cap on the accessory flagellum and a constricted inner ramus on uropod 2.

*Thrombasia* was originally erected for *Thrombasia tracalero* J.L. Barnard, 1966, and a diagnosis was provided for the monotypic genus, which was compared to a group of ‘ambasia genera’ (J.L. Barnard 1966). *Thrombasia* was later synonymised with *Schisturella* by Barnard and Karaman (1991). In 2014, the genus *Thrombasia* was re-established based on the gnathopod 1 coxa, which is tapering and slightly reduced, not vestigial as in *Schisturella*, and three new species were described (Kilgallen and Lowry 2014). The genus is distributed in the Pacific Ocean and South Atlantic Ocean: *T. evalina* Kilgallen & Lowry, 2014, from Tasman Sea, Australia, 820–923 m; *T. grabenis* (J.L. Barnard, 1967), East Pacific Ocean, southwest of Cedros Island, Mexico, 1720–1748 m; *T. rotundata* (K.H. Barnard, 1925), South Atlantic Ocean, Cape Point, South Africa, 1189–4050 m; *T. saros* Kilgallen & Lowry, 2014, Bass Strait, Australia, 1840 m; *T. tracalero* J.L. Barnard, 1966, East Pacific Ocean, California, USA, 167–183 m; *T. umina* Kilgallen & Lowry, 2014, Tasman Sea, Australia, 896–1066 m.

Here we add a new species to the genus *Thrombasia* collected from the Clarion-Clipperton Zone at depths of 4340–4356 m, which is an extension to the depth distribution for the genus, and provide an amended diagnosis of the genus and a key to its seven species. We also provide the first molecular barcodes for the genus.

## Material and methods

The material for the present study was sampled in the central-east Pacific Ocean, specifically in the easternmost sector of the Clarion-Clipperton Zone (CCZ). The material was collected using an epibenthic sledge (EBS) during two expeditions to the BGR exploration contract area (henceforth, contract area) in the CCZ; MANGAN 2016 (Rühlemann et al. 2017) and MANGAN 2018 (Rühlemann et al. 2019). For details of gear types and sample processing, see the relevant cruise reports, Jażdżewska and Horton (2026).

The habitus of the paratype male specimen SMF 63365 is presented as a photograph obtained with a confocal laser scanning microscope (CLSM). The specimen was stained in Congo red and acid fuchsin, temporarily mounted onto slides with glycerin and examined with a Leica TCS SPV equipped with a Leica DM5000 B upright microscope and three visible-light lasers (DPSS 10 mW 561 nm; HeNe 10 mW 633 nm; Ar 100 mW 458, 476, 488 and 514 nm), combined with the software LAS AF v. 2.2.1 (Leica Application Suite, Advanced Fluorescence). A series of photographic stacks were obtained, collecting overlapping optical sections throughout the whole preparation (Michels and Büntzow 2010; Kamanli et al. 2017).

The holotype specimen was dissected and mounted onto permanent slides using polyvinyl-lactophenol stained with lignin pink. Illustrations were made using Nikon SMZ1500, or Nikon Eclipse Ci microscopes equipped with a camera lucida. Pencil drawings were scanned and inked digitally using Adobe Illustrator and a WACOM digitiser tablet (Coleman 2003, 2009). Some setae are omitted from the illustrations for clarity. Appendages of the left side are dissected and illustrated unless otherwise stated.

In the descriptions and figures the following abbreviations were used: **A1, A2** = antenna 1, 2; **E1–E3** = epimera 1–3; **Ep** = epistome; **G1, G2** = gnathopod 1, 2; **H =** head; **LL** = lower lip; **Md** = mandible; **Mx1, Mx2** = maxilla 1, 2; **Mxp** = maxilliped; **P3–P7** = pereopod 3–7; **T** = telson; **U1–U3** = uropod 1–3; **UL** = upper lip; **l** = left; **r** = right.

The registered type material is deposited in the Senckenberg Museum (SMF; Frankfurt, Germany). All the remaining material is kept at the Deutsches Zentrum für Marine Biodiversitätsforschung (DZMB) in Wilhelmshaven.

All individuals were subjected to cytochrome *c* oxidase subunit I gene (COI) barcoding prior to identification of the species following methods presented in Jażdżewska et al. (2025). The relevant voucher information, taxonomic classifications and sequences are deposited in the data set “DS-AMPHICCZ” in the Barcode of Life Data System (BOLD) (https://doi.org/10.5883/DS-AMPHICCZ) (www.boldsystems.org) (Ratnasingham and Hebert 2007).

## Results

### Systematics

**Order AMPHIPODA Latreille, 1816**

**Suborder AMPHILOCHIDEA Boeck, 1871**

**Superfamily LYSIANASSOIDEA Dana, 1849**

#### Family TRYPHOSIDAE

Taxon classificationAnimaliaAmphipodaTryphosidae

Lowry & Stoddart, 1997

4057B54A-C303-5D36-AD25-F8FF37DE86EF

Thrombasia J.L. Barnard, 1966
Thrombasia J.L. Barnard, 1966: 72. ― Ledoyer 1986: 810. Schisturella. ―Barnard and Karaman 1991: 526. (in part) ― Kilgallen and Lowry 2014: 525.

##### Type species.

*Thrombasia tracalero* J.L. Barnard, 1966 (original designation).

##### Included species.

*Thrombasia* includes seven species: *T. ania* sp. nov.; *T. evalina* Kilgallen & Lowry, 2014; *T. grabenis* (J.L. Barnard, 1967); *T. rotundata* (K.H. Barnard, 1925); *T. saros* Kilgallen & Lowry, 2014; *T. tracalero* J.L. Barnard, 1966; *T. umina* Kilgallen & Lowry, 2014.

##### Diagnosis

(after Kilgallen and Lowry 2014). Antenna 1 flagellum article 1 lacking robust seta on distal margin; accessory flagellum forming cap. Antenna 2 flagellum articles 3–5 slender in female, article 3 enlarged in male; articles 3–5 with brush setae on the anterior margin. Mandibular incisor curved, smooth; molar a reduced column with convex triturating surface or proximally setose and distally triturating; palp attached midway. Maxilla 1 ST-7 serrate along the distomedial medial margin; ST-D slender, apically cuspidate. Maxilliped outer plate apical robust setae present. Gnathopod 1 subchelate; coxa slightly to greatly shorter than coxa 2, tapering distally; carpus slightly longer than propodus; propodus palm acute, straight. Pereopod 4 coxa with well-developed posteroventral lobe. Uropod 2 inner ramus constricted. Uropod 3 rami plumose setae absent in female, present occasionally in adult male. Telson moderately cleft.

##### Remarks.

The genus *Thrombasia* was established by J.L. Barnard (1966) for *T. tracalero* and was subsequently placed in the synonymy of *Schisturella* by Barnard and Karaman (1991). Kilgallen and Lowry (2014) revived the genus, considering *Thrombasia* to be distinct from *Schisturella* based on the gnathopod 1 coxa, which is tapering and slightly reduced, not vestigial as in *Schisturella*.

##### Distribution.

Pacific Ocean, South Atlantic Ocean.

#### Thrombasia ania sp. nov.

Taxon classificationAnimaliaAmphipodaTryphosidae

3E917B09-E659-56FB-A50B-FAB77F56EF39

https://zoobank.org/A3C3DA5F-5E05-4B50-BC81-CB553B05C264

Figs 1, 2, 3, 4, 5

##### Type material.

***Holotype***: Pacific • male, 3.5 mm, carcass and three slides; Clarion-Clipperton Zone; 11.791°N, 117.537°W; depth 4352 m; 09/05/2018; BGR contract area, RV "Sonne", Cruise MANGAN 2018, Station SO 262-155, epibenthic sledge; SMF 63365. ***Paratype***: PACIFIC • sex unknown, (damaged) ~4.5 mm, carcass and one slide; Clarion-Clipperton Zone; 11.791°N, 117.537°W; depth 4352 m; 09/05/2018; BGR contract area, RV "Sonne", Cruise MANGAN 2018, Station SO 262-155, epibenthic sledge; SMF 63366; • male, 5 mm, used for CLSM; Clarion-Clipperton Zone; 11.823°N, 117.544°W; depth 4340 m; 09/05/2018; BGR contract area, RV "Sonne", Cruise MANGAN 2018, Station SO 262-156, epibenthic sledge; SMF 63367.

##### Other material.

Pacific • unsexed (head only), not measured; Clarion-Clipperton Zone; 11.83°N, 117.508°W; depth 4344 m; 09/05/2016; BGR contract area, RV "Kilo Moana", Cruise MANGAN 2016, Station Ma 16-91, epibenthic sledge; DSB_3678; • unsexed, 2 mm; Clarion-Clipperton Zone; 11.798°N, 117.511°W; depth 4356 m; 09/05/2016; BGR contract area, RV "Kilo Moana", Cruise MANGAN 2016, Station Ma 16-95, epibenthic sledge; DSB_3679 • immature male, (damaged) ~3 mm; Clarion-Clipperton Zone; 11.791°N, 117.537°W; depth 4352 m; 09/05/2018; BGR contract area, RV "Sonne", Cruise MANGAN 2018, Station SO262-155, epibenthic sledge; DSB_3611 • unsexed, 2.5 mm; Clarion-Clipperton Zone; 11.823°N, 117.544°W; depth 4340 m; 09/05/2018; BGR contract area, RV "Sonne", Cruise MANGAN 2018, Station SO 262-156, epibenthic sledge; DSB_3615 • unsexed, 2 mm; Clarion-Clipperton Zone; 11.823°N, 117.544°W; depth 4340 m; 09/05/2018; BGR contract area, RV "Sonne", Cruise MANGAN 2018, Station SO 262-156, epibenthic sledge; DSB_3617 • unsexed, 2.5 mm; Clarion-Clipperton Zone; 11.823°N, 117.544°W; depth 4340 m; 09/05/2018; BGR contract area, RV "Sonne", Cruise MANGAN 2018, Station SO 262-156, epibenthic sledge; DSB_3618.

##### Type locality.

Abyssal Pacific Ocean, Clarion-Clipperton Zone; 11.791°N, 117.537°W; depth 4352 m.

##### Diagnosis.

Lateral cephalic lobe broadly triangular, apically subacute; process of upper lip broadly rounded and weakly protruding in front of epistome; coxa 1 weakly tapering, broad, about as long as coxa 2; gnathopod 1 propodus with acute palm, palm margin slightly concave; gnathopod 2 carpus length 1.6× propodus; coxa 4 posteroventral lobe rounded; pereopod 7 basis length 1.7× width; epimeron 3 posteroventral corner acute, slightly produced; uropod 2 inner ramus, spine at constriction not reaching end of ramus; uropod 3 outer ramus, article 2 long, length 0.9× article 1; telson short, length 1.2× width, cleft 29%, lobes closely appressed.

##### Description.

Based on holotype male, 3.5 mm SMF 63365; and paratypes SMF 63367 and SMF 63366, where indicated.

**Figure 1.**
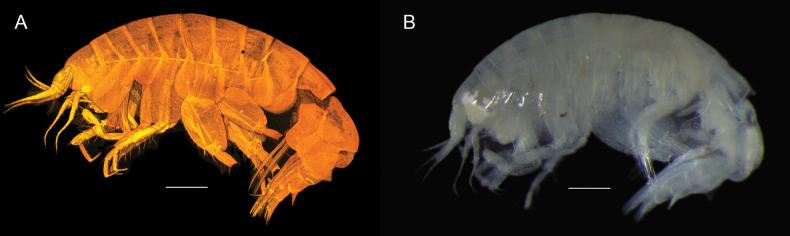
**A**CLSM photograph of *Thrombasia ania* sp. nov. **B** microscope photograph of *Thrombasia ania* sp. nov.; both habitus, male, 5.0 mm, paratype SMF 63367. Photograph by Anna Jażdżewska. Scale bars: 0.5 mm.

**Figure 2.**
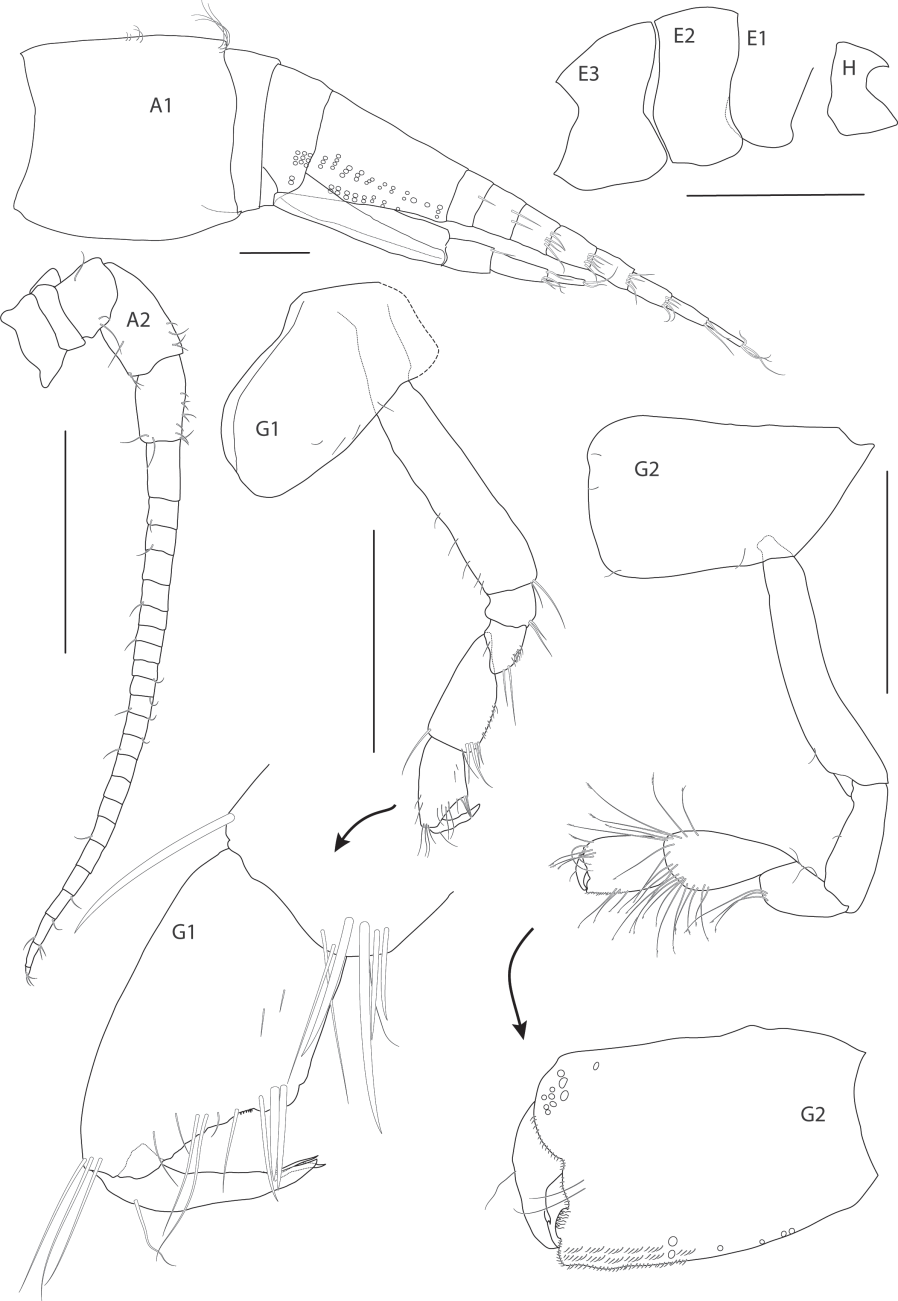
*Thrombasia ania* sp. nov., male, 3.5 mm, holotype SMF 63365. Scale bars: 1 mm (**E1–E3, H**); 0.1 mm (**A1**); 0.5 mm (**A2, G1, G2**).

**Body** (Figs 1, 2): ***Pereonites*** 1–7 (Fig. 1A) smooth, deeper than long, successively longer. ***Pleonite 3*** (Fig. 1) with a rounded, posterodorsal elevation slightly overhanging urosomite 1. ***Urosomite 1*** (Fig. 1A) proximally broadly rounded, with a middorsal concavity. ***Urosomite 2*** (Fig. 1A) short, but not telescoped under urosomite 1. ***Epimeron 1*** (Figs 1A, 2): quadrate, anterodistal corner slightly narrow, posterior margin broadly rounded. ***Epimeron 2*** (Figs 1A, 2) subquadrate, anterodistal corner rounded, distal margin convex, posterodistal corner not produced, posterior margin straight. ***Epimeron 3*** (Figs 1A, 2) anterodistal corner rounded, ventral margin convex, posterodistal corner slightly prolonged acutely into a broad tooth, posterior margin slightly convex. ***Coxae 1–4*** (Figs 1A, 2) longer than corresponding pereonites, progressively longer, coxa 1 subequal to coxa 2, slightly tapered distally.

**Head** (Figs 1, 2): subequal in length to pereonites 1–2; rostrum short, not reaching half of lateral cephalic lobe. ***Lateral cephalic lobe*** (Figs 1A, 1B, 2) broadly triangular, subacute. ***Eye*** (Fig. 1B) appearing white in fresh specimen, but not apparent in preserved specimens, non-ommatidial, formed of pigment granules; somewhat pear-shaped and occupying much of the lateral cephalic lobe, extending up to antenna 1 insertion. ***Antenna 1*** short, length 0.2× body; peduncular article 1 dilated, length 1.2× width (SMF 63367 = 1.5×), lacking dorsal keel; peduncular articles 2–3 short; flagellum nine-articulate, with small setae, first article of flagellum callynophorate, furnished medially with double row of aesthetascs; accessory flagellum four-articulate, first article broader and longer than remaining articles combined, calceoli absent. ***Antenna 2*** approximately equal to antenna 1, gland cone small: peduncular article 4 longer than 5, with short posteromedial setae; flagellum 22–23-articulate, calceoli absent.

**Figure 3.**
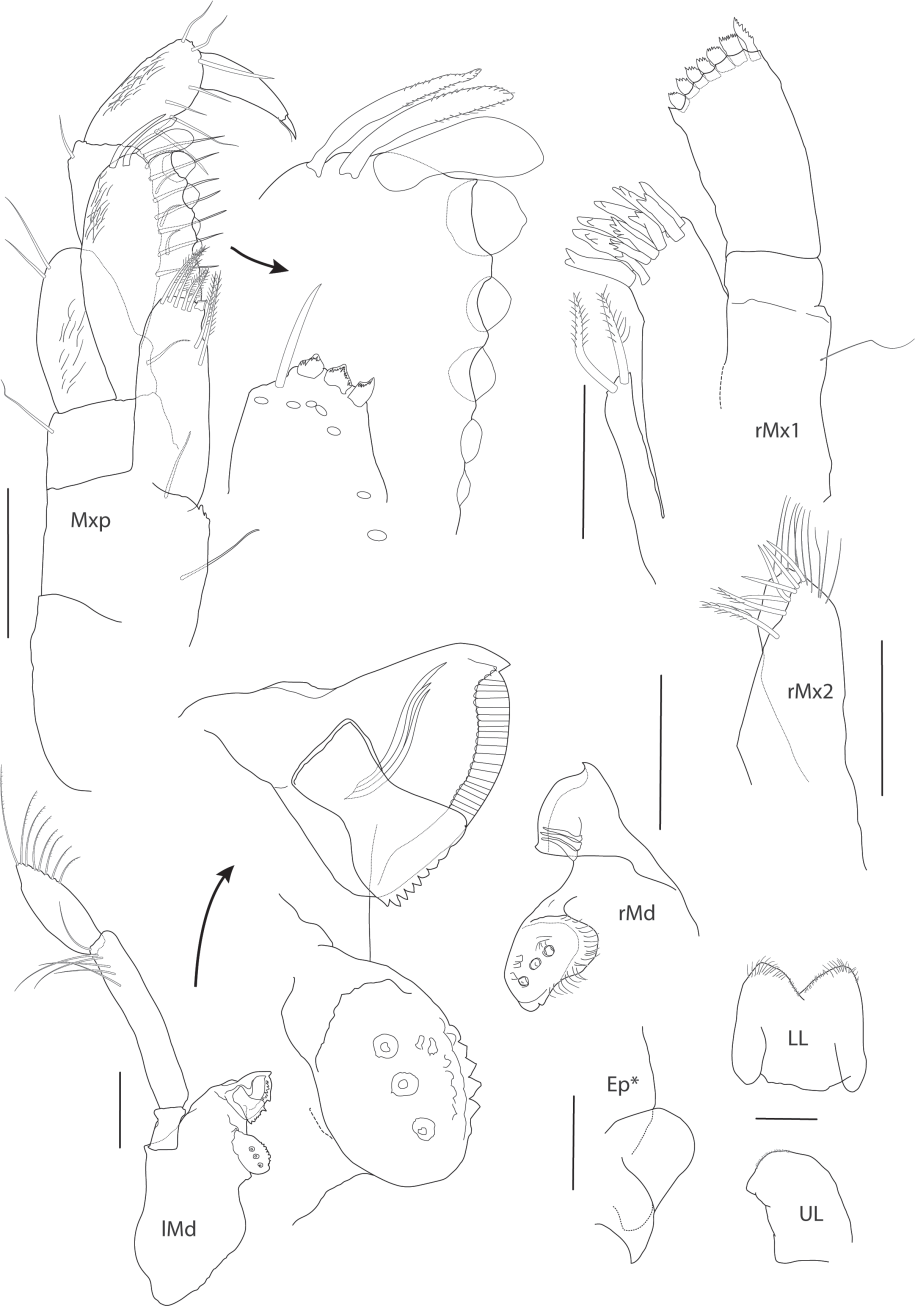
*Thrombasia ania* sp. nov., male, 3.5 mm, holotype SMF 63365. Scale bars: Mx1, Mx2, Mxp, lMd, rMd, UL, LL, Ep. *Paratype SMF 63366: 0.1 mm.

**Mouthparts** (Fig. 3): ***Epistome*** (of paratype SMF 63366) weakly convex. ***Upper lip*** process broadly rounded and weakly protruding in front of epistome, ventral margin rounded, with fine setules. ***Mandible*** incisor convex and widened, with tooth at anterodistal and posterodistal corners; left lacinia mobilis serrate, about 8-dentate, right lacinia mobilis lacking; left accessory spine row broken, right with three spines; molar ovate and strongly triturative, margin ridged, surface with three pits; palp attached level with molar, article 2 1.7× length of article 3, with five A2-setae, article 3 narrowly ovate, 0.6× length of article 2, with one A3, seven D3-pectinate setae and two E3-setae. ***Lower lip*** outer lobes broad with margins setose; inner lobes lacking, mandibular lobes narrow, rounded. ***Maxilla 1*** inner plate rectangular, narrow with two stout apical plumose setae; outer plate broad, with 11 spine-teeth in 6/5 crown arrangement; palp 2-articulate, article 2 rectangular, with six stout contiguous serrated spines and one longer marginal spine. ***Maxilla 2*** inner plate slightly shorter and broader than outer tapering distally, both with row of pectinate medial marginal spines and setae. ***Maxilliped*** inner plate narrow, subrectangular, extending well past the distal end of the inner margin of palp article 1 and not reaching one-half of outer plate, distal margin with three nodular triangular spines and plumose setae; outer plate narrowly subovate, length 2.1× width, extending to the distal end of palp article 2, with three strong distal spatulate spines and five strong medial nodular spines; palp setose medially, article 2 longest, article 4 slightly shorter than article 3.

**Figure 4.**
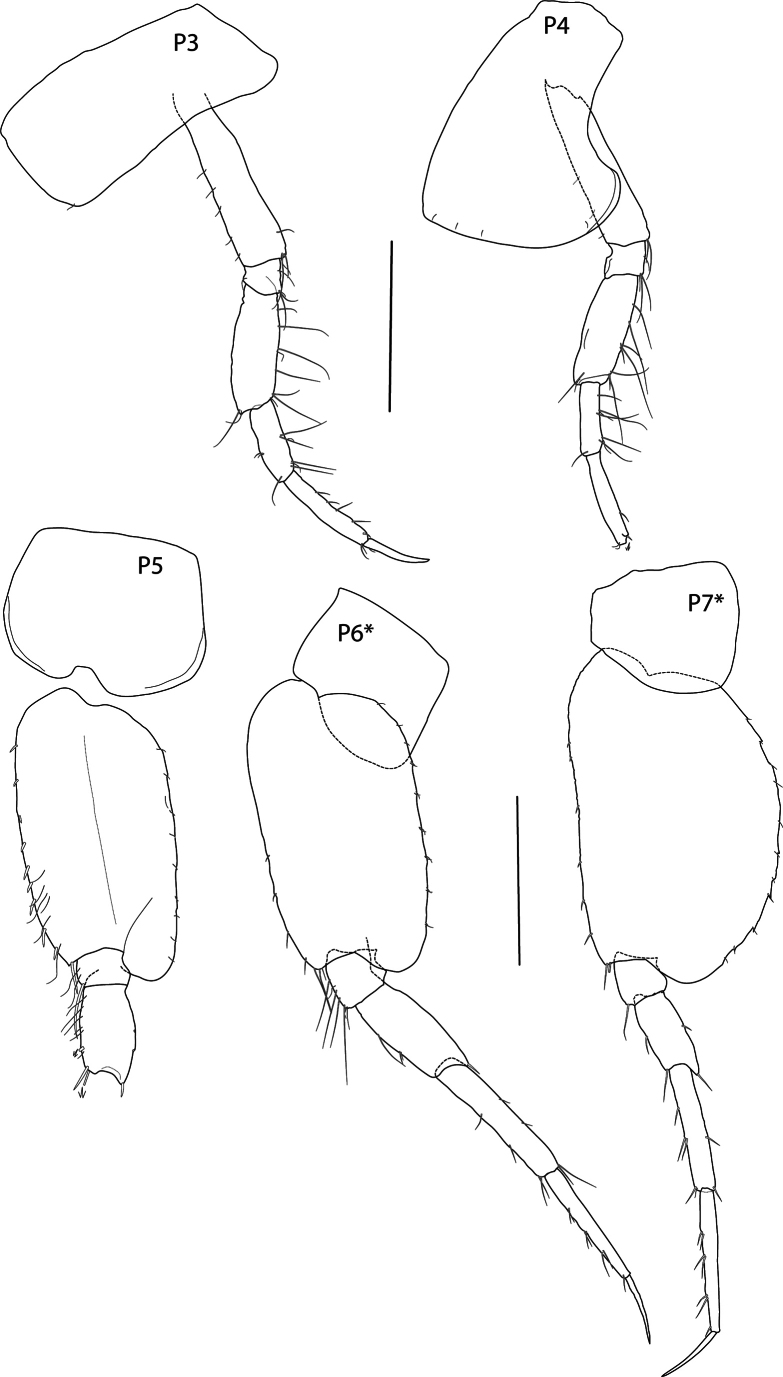
*Thrombasia ania* sp. nov., male, 3.5 mm, holotype SMF 63365. Scale bars: 0.5 mm (**P3–P7**). *Paratype SMF 63366.

**Pereon** (Figs 2, 4): ***Gnathopod 1*** (Fig. 2) coxa 1 weakly tapering, length 1.8× width, anterior margin slightly convex, anterodorsal corner rounded, posterior margin slightly convex, distal margin straight; basis, anterior margin with short setae distally; ischium subequal to merus; carpus without lobe, length 1.3× propodus, posterodistal margin setose; propodus short, length 1.7× width, subchelate, palm acute, slightly concave, palmar corner defined by two long spines; dactylus narrow, overriding palm corner. ***Gnathopod 2*** (Fig. 2) coxa rectangular, length 1.6× width; basis narrow, length 6.3× width, margins lacking setae; ischium longer than merus; carpus length 1.6× propodus, setose; propodus subovate, subchelate, with anterodistal groups of long pectinate setae, hind margin setose, palm short, nearly transverse; dactylus stout, not overriding palm corner. ***Pereopod 3*** (Fig. 4) coxa rectangular, with anterior margin very slightly convex, posterior margin slightly concave, length 2× width; merus longer than carpus, posterior margins with long setae; propodus longer than carpus, posterior margin weakly setose; dactylus straight, long, length 0.7× propodus. ***Pereopod 4*** (Fig. 4) coxa length 1.4× width, anterior margin convex, posterior margin deeply excavate proximally, with rounded posterodistal lobe located at distal 59% of the coxa length, ventral margin slightly convex; rest of pereopod articles as in pereopod 3 (dactylus missing). ***Pereopod 5*** (Fig. 4) coxa posterolobate, length 1.2× width, anterior and posterior margins rounded; basis, length 1.9× width, anterior margin spinose, posterior margin straight, slightly serrate, with narrow posterodistal lobe extending to or just beyond end of ischium; merus weakly expanded; remaining articles missing. ***Pereopod 6*** (of paratype SMF 63366) (Fig. 4) coxa posterolobate, subtriangular, posterior margin straight, lobe rounded; basis, length twice width, anterior margin straight with small spines distally, posterior margin straight with fine setules, posterodistal lobe just reaching end of ischium, ischium anterior margin with long setae; merus shorter than carpus; propodus narrow, shorter than carpus, with weak spines; dactylus slightly curved, length 0.5× propodus. ***Pereopod 7*** (of paratype SMF 63366) (Fig. 4) coxa posterolobate, subtriangular, posterodistally rounded; basis broadly expanded, length 1.7× width, anterior margin with weak spines distally, posterior margin convex, with small serrations and setules, posterodistal lobe just reaching end of ischium; merus-propodus as in pereopod 6 but narrower; propodus longer than carpus.

**Figure 5.**
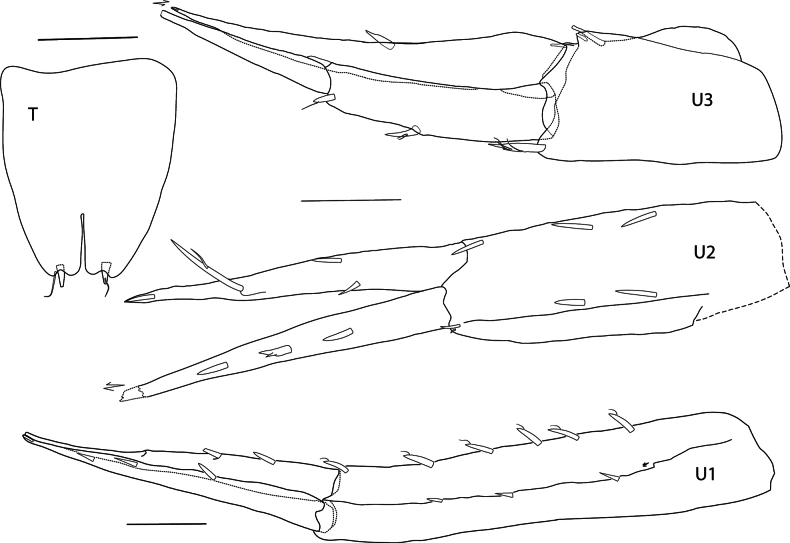
*Thrombasia ania* sp. nov., male, 3.5 mm, holotype SMF 63365. Scale bars: U1–U3, T: 0.1 mm.

**Urosome** (Fig. 5): ***Uropod 1*** peduncle length 1.4× rami, dorsolateral and dorsomedial margins spinose, with four and six spines respectively; rami lanceolate, equal in length, dorsolateral and dorsomedial margins with three spines. ***Uropod 2*** peduncle slightly shorter than outer ramus, dorsolateral and dorsomedial margins with two and three spines respectively; rami lanceolate with apical inset spine, inner ramus shorter than outer ramus, constricted, long spine at constriction not reaching end of ramus, dorsolateral and dorsomedial margins with one spine; outer ramus, dorsolateral margin with three spines, tip broken off. ***Uropod 3*** peduncle 0.6× length of biarticulate outer ramus, with one dorsomedial and distoventral spine; second article of outer ramus very long, 0.9× length of article 1, article 1 with two dorsolateral and one dorsomedial spine; inner ramus slightly shorter than outer, with one spine. ***Telson*** (Figs 1, 5) not reaching end of uropod 3 peduncle, short, length 1.2× width, cleft 29%, lobes closely appressed, with one apical spine in middle of lobe.

##### Etymology.

This species is named ‘*ania’*, used as a noun in apposition, in honour of Anna Jażdżewska, in recognition of her contribution to deep-sea amphipod taxonomy, and for the efforts she made to bring our ‘amphipod family’ together for the ISA SSKI workshop at the University of Lodz, Poland.

##### Remarks.

*Thrombasia ania* sp. nov. can be distinguished from all other species in the genus in having a small, broadly rounded process of the upper lip which scarcely protrudes in front of the epistome, and a long article 2 of uropod 3 (0.9× article 1). All other *Thrombasia* species have a long, produced lobe extending strongly in front of the epistome and a short article 2 of uropod 3 (less than 0.53× article 1).

*Thrombasia ania* sp. nov. has an upturned tooth on epimeron 3, which is found in three other species in the genus; *T. grabenis*, *T. tracalero* and *T. umina. Thrombasia ania* sp. nov. differs from *T. grabenis* in the uropod 2 incised spine length (longer in *T. grabenis* reaching the end of the ramus versus not reaching in *T. ania* sp. nov.), the outer ramus uropod 3 article 2 (longer in *T. ania* sp. nov. than in *T. grabenis*) and the telson which is more deeply cleft in *T. grabenis* (44% versus 29% in *T. ania* sp. nov.). The two species can also be separated by characters of the gnathopod 1 and 2. The maxilliped outer plate distal teeth are also more robust in *T. ania* sp. nov. than in *T. grabenis*.

The mandibular molar of *Thrombasia tracalero* is asymmetrical, proximally setose and distally triturating and thus differs from all other members of the genus which have a columnar molar with an oval fully triturating surface. *Thrombasia ania* sp. nov. also differs from *T. tracalero* in the shorter more shallowly cleft telson with closely appressed lobes (versus longer and more deeply cleft with apices separated in *T. tracalero*). The two species can also be separated by characters of the gnathopod 2.

*Thrombasia ania* sp. nov. can be separated from *T. umina* by the coxa 1 which is strongly tapering in *T. umina* and only weakly so in *T. ania* sp. nov. The gnathopod 1 palm is also more acute in *T. ania* sp. nov.

Only *Thrombasia rotundata* reaches abyssal depths where this new species is found, but that species is described from the South Atlantic. It also possesses a strongly protruding upper lip process (very weakly produced in *T. ania* sp. nov.), a short article 2 of uropod 3 outer ramus (very long in *T. ania* sp. nov.), a shorter truncated posterodistal lobe of coxa 4 (longer and broadly rounded in *T. ania* sp. nov.) and a narrowly rounded posterodistal corner of epimeron 3 (acutely produced in *T. ania* sp. nov.)

##### Distribution.

Abyssal Pacific Ocean, Clarion-Clipperton Zone, 4340–4356 m.

##### Molecular data.

Sequence data for the holotype of *Thrombasia ania* sp. nov. is deposited in GenBank under accession number PQ734300. The species has also received a Barcode Index Number from Barcode of Life Data Systems: BOLD:AEB6138 (https://doi.org/10.5883/BOLD:AEB6138).

### Key to the species of *Thrombasia*

**Table:** 

1	Epimeron 3 posterodistal corner produced into an upturned tooth	**2**
–	Epimeron 3 posterodistal corner broadly rounded, lacking tooth	**5**
2	Coxa 1 reduced, significantly shorter than coxa 2, tapering distally	**3**
–	Coxa 1 not reduced, about as long as coxa 2, weakly tapering distally	**4**
3	Telson length 1.3× width, cleft 45%, tapering distally	***T. umina***
–	Telson length 1.87× width, cleft 52%, lobes diverging	***T. tracalero***
4	Uropod 3 outer ramus, article 2 long, length 0.9× article 1; uropod 2 inner ramus spine at constriction short, not reaching end of ramus	***T. ania* sp. nov**.
–	Uropod 3 outer ramus, article 2 short, less than 0.6× article 1; uropod 2 inner ramus spine at constriction long, extending beyond end of ramus	***T. grabenis***
5	Coxa 1 long and weakly tapered	***T. saros***
–	Coxa 1 shortened and strongly tapered	**6**
6	Epimeron 3 posterodistal corner broadly rounded; gnathopod 1 palm straight	***T. evalina***
–	Epimeron 3 posterodistal corner prolonged and narrowly rounded; gnathopod 1 palm slightly convex	***T. rotundata***

## Supplementary Material

XML Treatment for TRYPHOSIDAE

XML Treatment for Thrombasia ania

## Additional information

### Author ORCIDs

Tammy Horton https://orcid.org/0000-0003-4250-1068

Georgina Valls Domedel https://orcid.org/0009-0005-8932-6641

Ed A. Hendrycks https://orcid.org/0000-0001-6199-7121

### Data availability

All of the data that support the findings of this study are available in the main text.
